# Symptomatology of Some Dental Lesions

**Published:** 1893-09

**Authors:** F. W. Ryan

**Affiliations:** Windsor, N. S.


ARTICLE III.

### SYMPTOMATOLOGY OF SOME DENTAL LESIONS.

BY F. W. RYAN, D. D. S., WINDSOR, N. S.

Pain is undoubtedly one of the most dreaded accompaniments of physical ills, and, as special practitioners of dental surgery, we are frequently asked to afford relief from this troublesome condition in and about the teeth. These pains may result from any one or more of a number of different causes, hence it becomes us as dentists to consider well the methods of determining as to which cause may be operating in any particular case.

The object of this paper, therefore, is not to suggest treatment, but is an endeavor to bring more prominently before us some of the symptoms peculiar to the different conditions, in order that we may proceed in a rational manner to adopt such measures as will bring about the required result.

As it is desired to be as practical as possible, we will not take into consideration those cases which rarely come under our observation, such as extensive inflammation of the maxillæ, necrosis, tumors, etc., but will confine our attention to such as regularly appear at our offices from day to day.

By far the greater amount of suffering we are called upon to treat results from lesions of the dental pulp or the peridental membrane, these being the two sensory organs of a tooth. The peridental membrane is the organ of touch, while the principal office of a normal dental pulp seems to be that of resentment to extreme degrees of temperature, and so, when we are asked why a certain sound tooth is a little sore to bite upon, we know that it is the organ of touch that is affected, probably with a slight inflammation which, in the absence of persistent irritation will usually yield to a little mild antiphlogistic treatment.

The pulp may, and does under other conditions, give rise to sensations when irritated by other than degrees of heat or cold. Sometimes a patient calls upon us, complaining of quite severe paroxysms of pain, induced by something sweet or sour coming in contact with a tooth that to the eye may appear quite intact, but the pain indicates that a change has taken place in the organic structure of the tooth that induces a separation of its organic and inorganic elements, which modification renders the former sensitive and the latter an easy prey to the acid secretions of the mouth.

We need not, therefore, be much surprised if after a few months our patient returns to us for further treatment, complaining, probably, of pain when masticating, or if particles of food becoming wedged between the teeth, and as these are difficult to remove, they are allowed to remain until fermentation increases their irritating properties, and pain is felt, though usually not severe. The tooth is not sore to touch, nor does it respond by unusual pain if cold be applied to its external surface; but allow the cold to be applied directly to the sensitive spot, then quite severe pain will follow. This would indicate superficial caries; but if the pain had been very severe and persistent, and recurring apparently without any exciting cause at intervals of some hours or days, we may expect more serious trouble—deep-seated caries, at least, if not pulp exposure, and in these cases it is often quite difficult to determine whether the pulp has been exposed or not.

The pain incident to an extensive exposure of dentine varies in degree with almost every individual case from those teeth which have never even given a feeling of discomfort to those which, upon the slightest provocation, or apparently without any provocation whatever, will give rise to the most violent paroxysms of pain.

What the peculiar conditions are that afford some of these cases immunity from suffering are not entirely clear. The irritability of the general nervous system has much to do with it, while the peculiar character of the teeth exerts a great influence. Those which are very hard, or have a large proportion of the inorganic elements, will not be found so sensitive as those in which the organic elements are at present in greater ratio. Hence the teeth of adults are not so sensitive as those of children. If the decay is slow, or if the vitality of the tooth be lost in advance of the decay of the inorganic elements, the sensitiveness will not be so great as would attend did the adverse condition prevail. An enumeration of all the agencies that conduce to this condition affords a good opportunity for research.

Deep-seated caries may often be ascertained by an intensified degree of the symptoms of the superficial caries, together with the absence of those which peculiarly designate an exposure of the pulp. Every practising dentist is well aware that the pulp may be, and often is, exposed without giving any warning of its condition, but in the great majority of cases when exposed, it has reached that degree of hyperæmia or inflammation which is its most abnormally sensitive condition. And we may expect such a condition if we are told that the patient has endured at irregular intervals a sense of undefined uneasiness in all the teeth of the affected side, more noticeable in the evening, and returning, perhaps, on successive evenings; that he has been awakened sometimes from a sound sleep, kept awake for an hour or two by throbbing pains in the tooth, then slept undisturbed for the rest of the night; that hot or cold water taken into the mouth is followed by severe pain which, perhaps, cannot be localized in a particular tooth, but seems to be on the surface of all the teeth; flashes of pain in the ear or the side of the face, or, perhaps, down the neck; in fact, a neuralgia which may be distinguished from the neuralgia of malarial or gouty origin, in that the paroxysms of the latter occur more frequently in the mornings instead of the evenings, and are not induced by bringing heat or cold in contact with the teeth, as is the case when its origin is to be found in the dental pulp.

If there be uncertainty as to which tooth is affected, it may be definitely ascertained by isolating each tooth in succession with the rubber dam, and applying the cold test until the affected tooth is reached which will respond by very severe pain.

It is true that a pulp does sometimes become more or less hyperæmic even in a sound tooth, the pain being sharp and lancinating, paroxysmal in its character, especially in the earlier stages, but in the absence of a cavity it is not easy to mistake this for any other affection, and if the tooth be protected from excessive thermal changes for a day or two, with perhaps a little topical application to the gums, the trouble usually passes away without any serious complications.

Sometimes it may be ascertained whether a pulp be exposed or not by cleaning the cavity somewhat and plugging with cotton and gum sandarac. If the pulp be not exposed the pain will gradually subside, otherwise it will continue unabated.

Not unfrequently, I think, those cases which sometimes return to us paining severely after being filled, are due to their having been filled over an unexpected exposure of the pulp when we thought we were contending only with dentine of exalted sensibility.

The differentiation of apical pericementitis presents little difficulty if we remember that the peridental membrane is the organ of touch for the tooth. Pain on pressure, therefore, is a constant symptom that distinguishes pericementitis from hyperæmia or inflammation of the dental pulp.

In pericementitis pain is always referred definitely to the affected tooth. In pulpitis the patient is usually uncertain as to the exact location of the pain. Reflected pains are characteristic of pulp trouble. They are not present in pericementitis. The pulp, especially when diseased, is very sensitive to thermal changes. The peridental membrane is not. Swelling that is apparent is uniformly absent in affections of the dental pulp; while in diseases of the peridental membrane swelling is usually present either in slight degree or extensively. These symptoms are sufficiently antithetical to make it easy to decide as to which of these two organs is affected.

The indications of chronic apical pericementitis are similar to those of the acute variety, but in modified form. Pressure on the tooth causes pain which may be considerable or only sufficient to cause annoyance. This condition may remain stationary, or it may last two or three days, then disappear for a time. There is more or less congestion over the root of the affected tooth and no sensitiveness to thermal changes.

If the means employed for subduing the inflammation have failed, or if the case be not seen until the formation of pus has began, all the symptoms will show aggravation.

The gums will become deeply congested or perhaps actually inflamed, and the lymphatics at the angle of the jaw are often sore and swollen. The pain in many instances becomes intolerable, and is of that throbbing, pulsing character that may be followed by rigor and high fever. After two or three days, or sometimes longer, the accumulated pus burrows through the bone and finds its way into the soft tissue where there is usually considerable swelling with a marked abatement of the intense pain. It continues, however, in a less intense form until the pus be discharged, when it subsides within a few hours, and the swelling within a day or two.

After the pus has been discharged from an acute abscess the inflammation subsides, and as far as the eye can detect the parts return to very nearly the natural condition, except that usually there remains a fistulous opening upon the gum, and now we will have a chronic alveolar abscess. Chronic alveolar abscess may also result without there having been any acute inflammation at any time. In fact, the patient may not remember that the tooth has ever been sore or that there ever was anything wrong with it. It may not even be decayed, but there is usually more or less discoloration, or if this be not perceptible, the particular tooth causing the trouble may be ascertained by the application of the cold test. This time the affected tooth will not give any response to cold while the healthy teeth on each side will be normally sensitive.

I do not consider it advisable to extend this paper so as to include the symptomatology of other affections of the oral cavity less frequently met with, nor do I submit the observations herein presented as at all exhaustive of this part of the subject, but if I have offered any suggestion, or if this paper is fortunate enough to induce a discussion that may suggest different methods of diagnosing these different affections, my object has been attained.—*Dominion Dental Journal.*

